# “It’s making me think outside the box at times”: a qualitative study of dynamic capabilities in surgical training

**DOI:** 10.1007/s10459-022-10170-2

**Published:** 2022-10-26

**Authors:** Adarsh P. Shah, Kim A. Walker, Kenneth G. Walker, Lorraine Hawick, Jennifer Cleland

**Affiliations:** 1 Centre for Healthcare Education Research and Innovation (CHERI), School of Medicine, Medical Sciences and Nutrition, University of Aberdeen, Aberdeen, AB25 2ZD UK; 2 NHS Education for Scotland, Centre for Health Science, Inverness, UK; 3 Lee Kong Chian School of Medicine, Nanyang Technological University, Singapore, Singapore

**Keywords:** COVID-19, Dynamic capabilities, Graduate medical education, Qualitative research, Surgical training, Training programs

## Abstract

Craft specialties such as surgery endured widespread disruption to postgraduate education and training during the pandemic. Despite the expansive literature on rapid adaptations and innovations, generalisability of these descriptions is limited by scarce use of theory-driven methods. In this research, we explored UK surgical trainees’ (*n* = 46) and consultant surgeons’ (trainers, *n* = 25) perceptions of how learning in clinical environments changed during a time of extreme uncertainty (2020/2021). Our ultimate goal was to identify new ideas that could shape post-pandemic surgical training. We conducted semi-structured virtual interviews with participants from a range of working/training environments across thirteen Health Boards in Scotland. Initial analysis of interview transcripts was inductive. Dynamic capabilities theory (how effectively an organisation uses its resources to respond to environmental changes) and its micro-foundations (sensing, seizing, reconfiguring) were used for subsequent theory-driven analysis. Findings demonstrate that surgical training responded dynamically and adapted to external and internal environmental uncertainty. Sensing threats and opportunities in the clinical environment prompted trainers’ institutions to seize new ways of working. Learners gained from reconfigured training opportunities (e.g., splitting operative cases between trainees), pan-surgical working (e.g., broader surgical exposure), redeployment (e.g., to medical specialties), collaborative working (working with new colleagues and in new ways) and supervision (shifting to online supervision). Our data foreground the human resource and structural reconfigurations, and technological innovations that effectively maintained surgical training during the pandemic, albeit in different ways. These adaptations and innovations could provide the foundations for enhancing surgical education and training in the post-pandemic era.

## Introduction

Change in medical education is notoriously difficult (Black, 2017; Katz, 2013; Mennin & Kaufman, 1989; Pock et al., 2019; Sood, 2008; Velthuis et al., 2021; Wartman, 2019). When change does happen such as in the case of curricular reforms, it is typically carefully considered, occurs in relatively stable environments (Harris et al., 2020), and takes a significant amount of time (Dougherty & Andreatta, 2017; Iobst et al., 2010; Long, 2000). However, curricular reforms under these circumstances often result in repetition of sameness with little actual reform (Hawick et al., 2017; Mennin, 2010; Whitehead et al., 2013).

In contrast, the COVID-19 pandemic-induced changes in medical education were reactive (Ajjawi & Eva, 2021), global rather than local (Papapanou et al., 2021; Rose, 2020; Wanigasooriya et al., 2021), set against a background of chaos, undertaken with unprecedented speed, and notable for innovation. Many rapid publications described how medical schools and residency/training providers “kept the [educational] show on the road” using technology (e.g., Ajjawi & Eva, 2021) and simulation. The majority of these papers were descriptive, with few providing outcome measures. Those that did, largely reported learners’ reactions to change–usually in terms of satisfaction with online and/or simulated learning (e.g., Daniel et al., 2021; Gordon et al., 2020; Grafton-Clarke et al., 2021). Very few studies used a theoretical or conceptual framework, thus limiting the generalisability of findings. Hence, while interesting, much research on adaptations during COVID-19 is limited in terms of its’ usefulness for enhancing medical education and training in the longer term. Theory-driven studies are needed to understand how system changes brought about by COVID-19 led to educational innovations, and how learners perceived these innovations.

We were specifically interested in the clinical learning environment in surgical training during COVID-19. Pre-existing tensions between service delivery and surgical training (Cleland et al., 2018; Marriott et al., 2011) were exacerbated by the pandemic (Lund et al., 2021). Surgical trainees (residents) in the UK and elsewhere experienced cancellation of elective operative lists, transformation of outpatient clinic encounters to virtual platfo2021arms, redeployment of trainees to medical specialties, and reorganisation of education and training activities (Clements et al., ; Dattani et al., 2020; Dedeilia et al., 2020; Hope et al., 2021; Khan et al., 2020; Lund et al., 2021; Munro et al., 2021; Rana et al., 2020; Research Education INnovation in Surgery (REINS) initiative COVID-19 et al. 2021). Consequently, trainees faced difficulties achieving their competency milestones (Aziz et al. 2021; Clements et al. 2021a, b; Dedeilia et al. 2020; Megaloikonomos et al. 2020; Osama et al. 2020). However, despite the reduction in working hours and a shift to online learning, trainees still experienced workplace learning, albeit in very different ways from the pre-pandemic norm. Moreover, anecdotal accounts suggested that while ‘normal’ learning opportunities were lost, new ones arose. Given this, our aim was to explore how the structures of learning changed in clinical environments during COVID-19, and how these adaptations were perceived by surgical trainees and their trainers (consultant surgeons). To do this, we turned to the management literature, specifically to the theory of Dynamic Capabilities (DC) (Eisenhardt & Martin, 2000). To the best of our knowledge, this is the first health professionals education study to use DC.

DC theory has a relatively long history and multiple definitions (Wang & Ahmed, 2007). We adopted Pavlou and El Sawy’s definition of DC as “those capabilities that help units extend, modify, and reconfigure their existing operational capabilities into new ones that better match the changing environment” as it was most apt for our research question and context (Pavlou & El Sawy, 2011). In short, DC can be thought of as how effectively an organisation can use its resources, or competencies (a term used in a very different way in the health professions education literature), including the tangible (e.g., equipment), intellectual (e.g., logos, research and development), and human resources to respond to internal and/or external environmental changes (Collis & Montgomery, 2008).

DC theory was developed for competitive industries, and posits that where the competitive landscape is shifting, the ways by which firm managers “integrate, build, and reconfigure internal and external competencies to rapidly address changing environments”(Teece et al., 1997)(p516) become the source of sustained competitive advantage. The (re)configuration of a firm’s competencies is what leads to dynamic capabilities (Ambrosini & Bowman, 2009; Lepak et al., 2007).

The DC framework is underpinned by three micro-foundations (Teece, 2007), which if successfully deployed lead to effective strategic change (Karali et al., 2018). These are sensing, seizing, and reconfiguring. Table 1 presents these micro-foundations and gives two illustrations of these during COVID-19, one from higher education and the second from a non-healthcare industry.

**Table 1:** Illustrative examples from higher education and non-healthcare industry of the three micro-foundations underpinned dynamic capabilities

Micro-foundation	Sensing	Seizing	Reconfiguring
Definition(Scoblic, 2020)	Requiring the perception and attention to sense opportunities and threats in the environment	The ability to take advantage of nascent opportunities, requiring flexibility in physical assets and organisational structures (agility)	The ability to recombine assets and restructure the organisation to match the environment
Higher Education(Fenech et al., 2021)	Qualitative study investigating how DC led to processes through which teachers created new teaching strategies when forced to transfer to online teaching in response to COVID-19		
	Challenges of student engagement and lack of face-to-face interaction with online learning Teachers’ personal learning opportunity in developing new skills from the shift to online teaching	Use features of the online platforms to engage students e.g., online polls, breakout groups, videos Mobilising resources to introduce gamification and simulations	Reforming teaching strategies according to course; becoming flexible and innovative Development of a repertoire of teaching strategies amongst teachers within the institution
Non-healthcare company(Puliga & Ponta, 2021)	An exploratory multiple case study of fast innovation reactions (masks, sanitizing gels, air cleaners, valves, and filtering screens) in seven non-healthcare companies that adapted their processes to cope with new and unexpected societal needs		
	Detecting the need for supply (external opportunity) and directing internal research and development toward problem solving and product development	Mobilisation of resources to develop the new product, fast and flexible decision making, and initiating combinatorial activities to meet supply demands	Capacity to use new and existing knowledge to maintain competitive advantage by developing different new products in response to the next turbulent market environment

Thus, using a Dynamic Capabilities lens to organise and interpret our data, we sought to uncover more about how COVID-19 adaptations in surgical service delivery impacted on learners. Our specific research question was: What are trainees and trainers’ experiences of workplace adaptations and innovations in response to the pandemic? By exploring the new resource and knowledge capabilities that may have been unlocked in surgical training during the first few months of the COVID-19 pandemic, our ultimate aim is to develop new ideas which can shape the future of surgical training.

## Methods

This qualitative study was underpinned by social constructionism, which acknowledges participants’ individual experiences and emphasises the social dimensions to meaning making (McMillan, 2015). We used individual interviews to identify and explore participants’ perceptions of opportunities in surgical training that have arisen as a result of altered or new ways of working.

### Context

After completing medical school in the UK, graduates undertake the generic, mandatory two-year Foundation Programme, then apply for specialty (residency) training (Rose & Aruparayil, 2021; Royal College of Surgeons of England, 2022). The first two years of surgical training, Core Surgical Training (CST) aims to deliver basic surgical skills and knowledge across the breadth of surgical specialties, thereby enabling trainees to make an informed sub-specialty career choice.

NHS Education for Scotland (NES) oversees the delivery and governance of CST in Scotland. This is organised into two programmes, East of Scotland (EoS) and West of Scotland (WoS), with a total intake of 40–50 core surgical trainees. Both programmes span a wide geographical area encompassing a variety of hospital settings, including regional tertiary units, district general and rural general hospitals. During every rotation, each trainee is assigned one educational supervisor, while clinical supervision can be undertaken by all consultant surgeons (“attendings”) within the department.

Since 2018, the EoS and WoS CST programmes have been subject to a Scotland-wide reform of early years surgical training called “Improving Surgical Training (IST)” (Allum, 2020; NES 2021; Royal College of Surgeons of England 2022). IST aims to improve the balance between service provision and training, improve trainee-trainer relationships by enhancing the quality of supervision, and advocate widespread use of simulation as an adjunct to surgical training through a fully funded simulation strategy (Blackhall et al., 2019; Walker & Shah, 2021; Walker et al., 2020).

In March 2020, the UK’s first national lockdown in response to the first wave of the COVID-19 pandemic significantly disrupted the delivery of routine healthcare and health professionals’ education, especially surgical training (Clements et al., 2021b; Dattani et al., 2020; Joyce et al., 2021; Khan et al., 2020; Lund et al., 2021; Munro et al., 2021). All surgical specialties were affected by the cessation of routine elective work and/or the reconfiguration of services/changes to operational policy to enable robust and safe provision of care that would withstand the peak of the pandemic (Editorial, 2021; El-Boghdadly et al., 2020). Face-to-face components of IST’s simulation strategy (courses, monthly training days) were either cancelled or modified to online learning, which took some time.

### Participants

On receiving project approval and appropriate institutional consents (see later), core surgical trainees and consultant surgeons from across Scotland were invited to take part in the study. The EoS and WoS TPDs emailed invitations to prospective participants on our behalf, between April 2020 and August 2020 (trainees and trainers) and February 2021 and May 2021 (trainers only). We also asked members of the team and research participants to assist us in identifying other potential subjects (snowball sampling (Parker et al., 2019)). During both participant recruitment rounds, email reminders about the study were sent out twice. Interested participants were asked to contact the main researcher directly by email. They were then provided with more information about the study and informed consent obtained.

### Data collection

We developed a semi-structured interview schedule (DiCicco-Bloom & Crabtree, 2006) informed by the literature (Gordon et al., 2020; Khan et al., 2020; Whelehan et al., 2021) and discussions both within the team and more widely with those involved in organising and delivering CST in Scotland. Interview questions were designed to explore participants’ positive and negative experiences of working within their department(s) and to ascertain institutional strategies implemented in dealing with the local effects of the pandemic. The questions also explored participants’ perceptions of the short-term and long-term implications of their observations and experiences on the future surgical training (Appendix 1).

The interview schedule ensured consistency, but interviews were iterative and continued until the participant felt he or she had shared their experiences sufficiently. As far as possible, open questions guided discussions, with prompts from the researcher to probe for deeper understanding of participants’ views. We continually reviewed the interview questions given the dynamic pandemic situation. AS conducted all interviews virtually using the Microsoft Teams platform.

### Data analysis

Interviews were digitally audio-recorded for later transcription and anonymised throughout the transcription process. Transcripts were entered into the qualitative data analysis software NVivo v12.0 (QRS International Pty Ltd, Doncaster, Victoria, Australia) to facilitate data management and coding. Coding occurred iteratively and inductively, focusing throughout on the research question. After team discussions of preliminary codes and resolution of any coding disagreements, we conducted a thematic analysis to identify themes and sub-themes (Ritchie & Lewis, 2013). After this, and following further team discussion, we extended beyond simple thematic analysis to critically analyse doctors’ perceptions of individual and institutional opportunities using the dynamic capabilities lens (Pavlou & El Sawy, 2011; Teece et al., 1997).

### Reflexivity

Qualitative research is dependent on the relationship between the researcher and the research process (Lincoln & Guba, 1985; McMillan, 2015). We considered our positions and relationships with the data continually and critically in view of our different inter-disciplinary backgrounds (psychology, pharmacology, nursing, and surgery), different levels of knowledge and experience of delivering and managing education, training, and research perspectives. For example, as a surgical trainee from another UK country who had taken time out of training to do a PhD, AS was both an insider and an outsider; external to Scotland’s healthcare system but an insider in terms of being a surgical trainee and having knowledge of training within the NHS.

### Ethics

The host University’s Research Governance team and the host NHS provider’s Quality Improvement and Assurance Team classified this study as a National Evaluation Audit (Project number 4945). This meant it was exempt from ethical approval. However, we followed core ethical principles: obtaining written, informed consent from potential research participants that their (anonymised) responses could be used for research purposes, that taking part was voluntary, and participants had the right to withdraw at any time.

## Results

Of the 91 trainees and 70 trainers invited to participate, 46 trainees and 25 trainers responded to the email invitation. Table 2 reports participant characteristics and the range of duration of the interviews. Thirteen Health Boards across Scotland were represented. Participants represented all surgical specialties except neurosurgery which does not have CST posts. They worked in a range of hospitals (*n* = 47) from urban tertiary centres to small district general hospitals, to remote and rural hospitals.

**Table 2:** Summary of the participant recruitment invitation outcomes, participants’ characteristics (by training programme location, gender, and training grade), and duration of the semi-structured interviews

		Trainees	Trainers
Recruitment email invitations	Total number of persons contacted	91	70
	Responded and agreed to interview	46	25
	Responded, but declined interview	3	0
	Responded, but failed to commit an interview date	4	9
	Did not respond	38	36
Training programme	East	26	14
	West	20	11
Gender	Male	23	19
	Female	23	6
Training grade	CT1	28	
	CT2	18	
Interview duration (minutes)	Total	2466	811
	Median	55	41
	Range	26– 111	18 – 60

Participants have been anonymised and identified as trainees (CT) or trainer (TR). We report verbatim quotes. An ellipsis (…) indicates text that has been cut out where less relevant, and square brackets indicate any non-verbatim explanatory text. There are different ways of presenting qualitative data: in this study, we have used the three micro-foundations of DC theory (sensing, seizing, and reconfiguring)(Teece, 2007) to organise the data and used quotations to aid confirmability. We present this data in the broad themes identified in our initial thematic analysis: training opportunities, pan-surgical working, redeployment, collaborative working, and supervision. Table 3 provides a summary of the results, and we elaborate on each theme below.

**Table 3:** Summary of results explaining the adaptations and reconfigurations in each theme against the relevant microfoundation

Microfoundation → Theme ↓	Sensing	Seizing	Reconfiguring
Definition(Scoblic, 2020)	Requiring the perception and attention to sense opportunities and threats in the environment	The ability to take advantage of nascent opportunities, requiring flexibility in physical assets and organisational structures (agility)	The ability to recombine assets and restructure the organisation to match the environment
Training opportunities	Cancellation or reduction of elective operating lists and clinic size COVID-19 added burden to existing complexity of patients	Increased trainer availability for supervision in theatre and clinic; enhanced opportunities for trainees Two-consultant operating model introduced	Maximising training by compartmentalising cases and conducting clinics jointly with trainees Altering rotas to pair trainees with trainers to gain maximum exposure
Pan-surgical working	The need to streamline patient flow	Exposure to wide range of surgical patients beneficial to trainees	Amalgamation of acute surgical services so patients from all surgical specialties triaged in one place
Redeployment	The need to provide additional support to non-surgical specialties in the face of increasing service demands	Opportunities for surgical trainees to learn new practical skills and knowledge	
Collaborative working		Working alongside new colleagues in dynamic teams and environments; individuals working in new roles within teams	Previously independent clinical and management teams working together
Supervision		Re-opening or re-structuring of physical spaces Moving to online methods for supervision increased trainee-trainer contact	Setting up a facility for simulation training

### Training opportunities

Patient attendances to out-patient clinics reduced dramatically during the early phases of the pandemic. This *reconfiguring* led to unique training opportunities: “we’ve reduced the size of clinics… so there would be potential to say, “okay, well I’ll separate the two halves of the clinic. You see them with me for more complex stuff,” and I think that’s quite educational” *(seizing)* (TR18).

The cancellation of elective operating lists afforded “all this fallow time where everything is slow” *(sensing)* (TR18), thereby the “opportunity [for trainers] to turn round and say, “take as long as you want” *(seizing)*” (TR18). Trainees reported being offered supervised, hands-on intra-operative training *(seizing)*: “…on the days that we only had one operation, he’d take me through it, and he would let me do it” (CT21). One trainer described ‘compartmentalising’ operative cases *(seizing and reconfiguring)*: “It is a strange thing to do is to split an operation up because it’s not how I was trained, you know, but it’s probably the right thing to do [with respect to providing maximum training opportunities] to split an operation up between trainees” (TR16).

Because of the added burden of COVID-19 to “the complexity of patients” (TR11) *(sensing)*, one vascular surgical department “instituted a two-consultant operating model” (TR11) *(seizing)*(Ellis et al., 2021)*.* This service delivery model was deemed conducive to training so rotas were altered to regularly pair trainees with trainers *(reconfiguring)*: “I’ve got feedback from the trainees that this is excellent. Because, for instance, if we’re doing a fem-pop bypass, one consultant will take the trainee through one end of the procedure and the other consultant will do the other half of the procedure…” (TR11).

### Pan-surgical working

Acute surgical services were amalgamated to streamline patient flow pathways to minimise unnecessary movement of patients *(sensing)*. Consequently, participants obtained pan-specialty exposure *(seizing)* from all surgical specialties *(reconfiguring)*: “…[patients negative for COVID-19 go] straight to a surgical assessment unit based on oh, you’ve got a head injury, you’re going into neurosurgery. And you’ve got a chest wall injury, you’re going to thoracic. So, we now see all of the patients that are coming through for any surgical subspecialty” (CT27). Participants valued this broad surgical exposure *(seizing):* “… we’re seeing things and assessing things and learning on the job… it’s making me a better generalist, and it’s making me think outside the box at times” (CT27).

### Redeployment

Core surgical trainees were redeployed to medical specialties, commonly critical care (intensive care medicine), gastroenterology, respiratory medicine, and emergency medicine to provide additional staff to cope with increasing service demands in those specialties *(sensing)*(Faria et al., 2020). This offered unique learning opportunities *(seizing)*: “…I haven’t done any critical care ever as part of my training. So, I’ve been able to put in central lines, arterial lines… I’ve found it very good to be able to have that experience of critical care and I think that’s helpful for me further down the line” (CT13). Similarly, another trainee’s redeployment to a medical ward complemented their parent surgical specialty *(seizing)*: “…she [trainee] was redeployed to the gastro[-enterology] ward, and because she was very proactive, she did lots of case-based stuff with the gastroenterologist on things like IBD [inflammatory bowel disease] and some of the liver stuff… that’s a side to upper GI that a lot of trainees never get to see, the kind of medical side” (TR22).

### Collaborative working

The fear of the unknown that accompanied the first wave of the pandemic brought increased collaborative working across clinical and management teams *(reconfiguring)*(Thornton, 2020). These structural changes were perceived as beneficial to trainees *(seizing)*: “having to work with colleagues that you’ve never worked with before, unusual environments, adaptation, resilience… those type of things, I think hopefully have benefitted the trainee” (TR09).

High infection rates amongst healthcare professionals depleted staffing levels, thereby leading to increased collaborative interprofessional teamworking: “we were doing procedures on the ward that needed nurse support, but because the nurses were not available, the FY1s and the FY2s were stepping in and out, and I was stepping in to give them a wee hand” *(seizing related to reconfiguring)* (CT21). Similarly, another participant reported “I think I’m a better team member now … when I go in and see a patient, I’ll happily do an ECG, dip their urine and you know, things that you would, you think, not were below you, but you just wouldn’t do them really” *(seizing)* (CT28).

### Supervision

Face-to-face supervisory activities ceased immediately because of the pandemic, so many trainers shifted supervision sessions to online *(seizing)* which actually led to more regular contact between trainers and trainees than was the case during normal pre-pandemic working: “My educational supervisor is fantastic. She actually took time, once a week, to just have a Teams conversation… and I used to choose a [case-based discussion] topic … I would have an hour where we would just talk about it…” (CT25). This situation opened up other opportunities *(seizing)*: “…Covid has allowed us to reopen one of our old wards and it gets multipurpose use and is now used as an EOsim [laparoscopic simulator] trainer room” (TR18) *(reconfiguring)*.

## Discussion

Using a Dynamic Capabilities lens to organise and interpret our data, we sought to identify and explore participants’ perceptions of opportunities in surgical training that occurred because of altered or new ways of working (Table 3). We identified some interesting modifications and reconfigurations which illustrated that, to paraphrase Winston Churchill, “the crisis was not allowed to go to waste”. First, less congested theatre lists afforded trainees’ opportunities to operate because time was less of an issue compared to normal working arrangements. Fewer patients meant trainers found innovative ways of arranging operating lists and clinics to ensure learning opportunities for multiple trainees were maximised. Trainees were exposed to more surgical and indeed medical specialties, allowing different opportunities for skills and knowledge development, as well as teamworking. Finally, the use of virtual supervision seemed to facilitate rather than hinder opportunities for trainee-trainer interaction.

Campaigns promoting surgical training during the pandemic (Lund, 2020) called for unconventional training opportunities. Evidence that this occurred is illustrated in our data (e.g., a dual consultant operating model and ‘compartmentalisation’ of operative cases for training). The latter is an interesting innovation that has seldom been described in the literature. Any incorporation of these dynamic capabilities as new routines (i.e., organisational learning) (Souza and Takahashi 2019) warrants further exploration as a means of maximising training opportunities during normal times.

The repurposing of spaces reported in our data facilitated simulation-based training during the pandemic. While reports of simulation-based teaching and learning innovations surged in response to diminishing practical training opportunities (e.g., Doulias et al., 2020; McKechnie et al., 2020; Naughton et al., 2021; Okland et al., 2020; Rawaf et al., 2022), our data adds that this resource reconfiguration also seemed to help prevent trainee isolation at a time when trainees were largely excluded from most operative activities (Whelehan et al., 2021).

Positive stories of harnessing the unique learning opportunities from redeployment within our data reflects reports from other settings (e.g., Sarpong et al., 2020; Seah, 2020). The multidisciplinary collaborative teamworking we report shares similarities to that observed in other contexts (e.g., Samuel, 2021). Our data also illustrates how trainees developed insight into allied health professionals and managers roles through collaborative working (Whelehan et al., 2021). Trainees also gained invaluable exposure to leadership and management skills (Doulias et al., 2020). The unexpected opportunities to develop these non-technical skills may be important in terms of performance, and ultimately patient safety (e.g.,Flin et al., 2016; Mazzocco et al., 2009; Way et al., 2003) in these cohorts of trainees.

Augmented training experiences during the pandemic highlighted in our data (e.g., virtual supervision facilitated regular meetings where trainees completed workplace-based assessments, reduced clinics enabled trainee attendance to complex patient clinics, and decreased elective workload freed up trainer time for supervision of simulated technical activities) were aligned to pre-pandemic IST aspirations. Thoughtful reflection is required to weigh up these gains versus other losses (i.e., regular supervision does not make up for inability to achieve technical competencies) and how to balance service with training as services and training recover from the pandemic.

More generally, the rapid and effective transition to online/virtual learning may herald a new era for surgical training (Doulias et al., 2020), facilitating access to learning in different localities. This is particularly relevant to countries such as Scotland where hospitals are spread out geographically. However, there is a balance to strike between virtual and face-to-face learning, given in-person education creates student-tutor and student–student interaction, which in turn promotes engagement with learning (Kunin et al., 2014). Moreover, one does not learn to be a doctor or surgeon by attending online classes: socialisation in clinical and educational settings is critical for professional identity formation and integration into the surgical community (Cleland et al., 2016; Nordquist et al., 2019).

Management science theory is rarely adopted in the surgical education literature (Cleland et al. (2018) is an exception). We carefully considered the DC theory and believed it was appropriate for our research question and context. For example, our focus on exploring the resource-related adaptations during the period of uncertainty was consistent with the DC theoretical framework (Eisenhardt & Martin, 2000; Pavlou & El Sawy, 2011; Teece, 2007). An exploration of the adaptions to surgical training structures was not the scope of our work, but future work could explore the relevance of the DC framework in, for example, rapidly reconfiguring assessment practices for postgraduate medical training during the pandemic (Health Education England 2021﻿).

The DC framework is not limited to periods of uncertainty and/or unstable external environments. It has been used in higher education to demonstrate routine activities with capabilities linked to innovation (Akram & Hilman, 2017). In other contexts, DC has been used to explain how and why organisations successfully adapt to changes in stable business environments (Kaur, 2019). While the pandemic-related restrictions to field work limited us in our data collection methodology, there is scope for employing DC in ethnographic studies to better understand planned change/innovation capabilities within health professionals education.

DC is a theory which was developed for competitive organisations, not public sector organisations, and there may be boundaries to its use with this setting and population. For example, DC is limited in its’ ability to examine tensions (Hayter & Cahoy, 2016). In saying that, the ‘sensing, seizing, and reconfiguring’ heuristic provided a simple yet effective way to elucidate the processes behind the strategies that were employed to adapt and maintain surgical training. This adds ‘behind the scenes’-type operational knowledge to bringing about rapid changes in medical education. Of course, DC theory illuminated certain aspects of the data (Bordage, 2009); another lens may have emphasised different aspects of the problem, such as the impact of reduced working and changes to training on the professional identity formation of surgeons in training, or emotional responses to not being able to achieve competencies as normal.

All qualitative data collection approaches have strengths and weaknesses (DiCicco-Bloom & Crabtree, 2006). Virtual interviews were the only way to obtain responses from trainers and trainees across many different contexts during the pandemic. It was difficult to recruit trainers initially, hence our second round of invitations to take part in the study. We believe this was due to the COVID-19 pandemic, so we waited until after (what was) the second wave of the pandemic before sending out second invitations to trainers only. As with any voluntary study, there would have been an element of participant self-selection. We did not decide the number of interviews in advance but rather collected interview data until a high degree of consensus had emerged and no new themes were apparent ( thematic saturation or sufficiency (Vasileiou et al., 2018)). Indeed, we had a large number of interviews from a reasonably homogenous population. The interviews were substantial in terms of length (Table 2) and rich data, so we feel confident that our data reflects common experiences. Our study design did not allow us to capture longitudinal processes (e.g., how things changed over time), or if experiences differed by stage of training or position. That said, the goal of the study was not to assess change longitudinally, but to explore the perceptions of the interviewees during a time of great upheaval.

## Conclusion

Our data foreground the human resource and structural reconfigurations, and technological innovations that effectively maintained surgical training during the pandemic, albeit in different ways from pre-pandemic times. Even in times of uncertainty, learners have benefited from the rapid adaptations and innovations that were hastily conjured to minimise disruption. We urge those involved in surgical training to consider how aspects of the innovations and reconfigurations introduced during the pandemic can be maintained, and properly evaluated, post-pandemic. It is critical not to automatically return to pre-pandemic norms, thereby missing an opportunity to implement change. Stepping back and looking at the positives which came out of the COVID-19 disruption to surgical training should encourage consideration of what to keep, and what resources are needed to facilitate change (Arroyo et al., 2021).

## Appendix 1

### Semi-structured interview questions: trainee

#### Introduction

Thank you for agreeing to participate in this study. Your point of view is important to us and your time is appreciated. This interview is being recorded and will be transcribed. It will be anonymised. Any personal or patient identifiable information that you accidentally volunteer will be anonymised or blanked. You are free to withdraw at any point during the interview and the recording will be deleted. The purpose of this interview is to discuss your training experiences within the IST pilot and the effect of COVID on those training experiences.

You are currently a CT1/CT2 *(select as appropriate).* Could you please inform me the hospitals and rotations that you have worked in?

#### Questions pertaining to training

I’d like you to think of the last *(start of core training)* months of your training, prior to and during the pandemic. Please describe your experiences of surgical training.

- Rotations
- Training opportunities offered
- Direct observation from supervisor
- Quality and quantity of operative exposure (60% elective work)
- Training and supervision in wards and clinic
- Emergency exposure
- Breadth and depth of specialty exposure
- Meeting curriculum requirements

Do you feel you have gained the core curricular competencies?

If yes, was there any one area/thing that helped with this? If no, why not.

Do you know what you want to specialise in?

Do you feel adequately prepared for Higher Specialty Training?

If you were asked to step up as the surgical registrar, how confident would you feel about this role? Explain why…

#### Questions pertaining to simulation

What access to simulation do you have in your training?

Has simulation assisted your surgical training? If so, how?

How do you think the simulation programme aligns with the curriculum?

#### Questions pertaining to supervision

How do you get on with your trainers?

What has made a difference in terms of developing a relationship with your trainer? (E.g. working together on a difficult op, writing a paper together).

Do you feel your clinical and educational supervisors are aware of your individual needs? How have they identified and addressed these?

Typically, what is your strategy for achieving your workplace-based assessments?

What has your educational supervisor done to support you?

#### Questions pertaining to institutional set up

What is in place by your department/institution to facilitate your training?

At your hospital, do you work within an extended surgical team? ***Describe the team*** (Advanced clinical practitioners, physician associates, surgical first assistants, surgical care practitioners).

If so, how does working in an extended surgical team influence your training?

#### Questions pertaining to overall impressions

How satisfied are you in your job?

Describe examples of your positive training experiences.

Describe examples of your negative training experiences.

What did you expect from IST? Is it what you expected? If not, why/how not?

Were you aware of any problems or challenges with surgical training leading up to IST? If yes, how do you think this new curriculum has helped?

#### Questions pertaining to COVID pandemic

Finally, I’d like you to reflect on events in the past few weeks. How has COVID impacted on your training? If so, how can you see any positives out of this? Any anxieties?

Do you think you will be able to get any WBAs done whilst you are working in another specialty?

Do you have an educational supervisor in your current position?

Are there any different cultures/working patterns that you would wish to take back to your surgical training?

I have asked all my questions. Is there anything else you would like to tell me either about your surgical training or the COVID19 situation?

## Semi-structured interview questions: Trainer

### Introduction

Thank you for agreeing to participate in this study. Your point of view is important to us and your time is appreciated. This interview is being recorded and will be transcribed. It will be anonymised. Any personal or patient identifiable information that you accidentally volunteer will be anonymised or blanked. You are free to withdraw at any point during the interview and the recording will be deleted. The purpose of this interview is to discuss your experiences of supervision, coaching and mentoring within the Scottish IST pilot.

#### Questions pertaining to supervisory role

How many trainees are you assigned a supervisor for? Of these, how many are core trainees?

How long have you been a clinical/educational supervisor for? Why do you undertake this role?

What do you know about IST?

How did you become familiar with the core surgical curriculum? How do you keep up with proposed changes to the surgical curriculum?

IST is currently in its third year. Please describe your experiences of mentoring and supervising IST trainees over the last *(timeframe relevant to participant)* months:

- Induction
- Supervisory meetings and reports
- Objective-setting / goal-setting
- Placement consolidation meetings
- Elective-emergency workload balance of trainees

How do these experiences compare with pre-IST trainees? (with respect to 2-weekly meetings, quality and quantity of training, access to simulation).

What are your expectations of IST trainees?

What helps in developing a relationship with the trainee?

In an average month, how often would you work with your assigned trainee?

- Theatres, wards, clinics, MDT, endoscopy
- Describe how you coach and supervise them in your clinical practice?
- What, in your opinion, is working well?
- What could be done better?

How do you go about completing trainees’ workplace-based assessments?

In your opinion, whose responsibility is it to set up the 2-weekly meetings?

How do you keep up to date with trainees’ progress (and portfolio)?

#### Questions pertaining to simulation

What access to simulation do your core trainees have?

Does simulation assist your core trainees? If so, how?

How do you think the simulation programme aligns with the curriculum?

#### Questions pertaining to institutional set up

What has your department/institution put in place to facilitate IST?

Where on the business agenda does training feature in your department/institution?

How has your Trust altered your job plan to facilitate your supervisor role?

How does your Trust reward you for the supervisory role you undertake?

At your hospital, do you work within an extended surgical team? ***Describe the team*** (Advanced clinical practitioners, physician associates, surgical first assistants, surgical care practitioners).

If so, how does working in an extended surgical team influence IST? How does this affect your role as supervisor? (increased workload, supervisor to EST therefore less time for CT?)

What support did you receive from NES regarding this role?

#### Questions pertaining to overall impressions

How satisfied are you in your role as supervisor?

Describe examples of your positive experiences of supervision.

Describe examples of your negative experiences of supervision.

What did you expect from IST? Is it what you expected? If not, why/how not?

#### Questions pertaining to COVID pandemic (*If not already discussed)*

Finally, I’d like you to reflect on events in the past few weeks.

How do your trainee’s current experiences compare to those pre-pandemic?

How has COVID impacted on your role as supervisor? If so, how can you see any positives out of this? Any anxieties?

How has your institution/department adapted to the disruption?

Are there any different cultures/working patterns that you think would be beneficial to surgical training?

I have asked all my questions. Is there anything else you would like to tell me either about your surgical training or the COVID19 situation?
